# Hamstring Strain Injury Risk in Soccer: An Exploratory, Hypothesis-Generating Prediction Model

**DOI:** 10.3390/muscles4040050

**Published:** 2025-11-04

**Authors:** Afxentios Kekelekis, Rabiu Muazu Musa, Pantelis T. Nikolaidis, Filipe Manuel Clemente, Eleftherios Kellis

**Affiliations:** 1 Laboratory of Neuromechanics, School of Physical Education and Sport Science at Serres, Aristotle University of Thessaloniki, 62100 Serres, Greece; ekellis@phed-sr.auth.gr; 2 Sport Injury Clinic for Prevention & Rehabilitation, 72100 Aghios Nikolaos, Greece; 3 Centre for Fundamental and Continuing Education, Universiti Malaysia Terengganu, Kuala Terengganu 21030, Malaysia; rabiumuazu86@gmail.com; 4 School of Health and Caring Sciences, University of West Attica, 12243 Athens, Greece; pademil@hotmail.com; 5 Gdansk University of Physical Education and Sport, 80-336 Gdańsk, Poland; filipe.clemente5@gmail.com

**Keywords:** hamstring injury, prediction model, logistic regression with elastic-net penalty model, machine learning, TRIPOD, soccer

## Abstract

Hamstring strain injuries (HSI) are common in soccer and remain challenging to predict, as traditional risk factors often fail to capture the multifactorial nature of injury susceptibility. This prospective cohort study aimed to develop and internally validate a machine learning-assisted logistic regression model for predicting hamstring injuries in amateur soccer players using preseason clinical and strength-related variables. A total of 120 male players were followed for one competitive season (30 weeks). Baseline predictors included age, body mass index, previous injury, and bilateral isometric hip and knee strength measured via handheld dynamometry. Twenty initial predictors were reduced to ten through symmetrical uncertainty feature ranking before training a logistic regression model with elastic-net regularization (training set: *n* = 83; test set: *n* = 37) using nested four-fold cross-validation. Model performance was evaluated using the area under the receiver operating characteristic curve (AUC), calibration metrics, and confusion matrices. During follow-up, 21 players sustained at least one HSI (32 events; 28% reinjuries), yielding an events-per-variable ratio of 2.1, below ideal thresholds and suggesting possible overfitting. On the independent test set, the model achieved an accuracy of 64.9%, AUC of 0.68 (95% CI 0.52–0.84), calibration slope of 0.85, and intercept of −0.12, with a sensitivity of 60% and specificity of 65.6%. Dominant-leg hip abduction strength was the only statistically significant predictor (OR = 0.82, 95% CI 0.70–0.96), while permutation importance analyses identified previous hamstring injury as the most stable contributor to model performance. Neither age nor hamstring isometric strength demonstrated predictive value. Although model discrimination was moderate and calibration indicated mild overfitting, findings reinforce the prognostic relevance of prior injury and suggest that reduced hip abduction strength may serve as an emerging candidate marker. This study, classified as a TRIPOD Category 2 model (development without external validation), provides preliminary, hypothesis-generating evidence supporting the use of multivariate strength and history-based predictors in future, larger-scale injury prediction research.

## 1. Introduction

Hamstring strain injury remains prevalent across sporting activities that involve sprinting, jumping, acceleration, deceleration, and rapid change in direction, resulting in significant time-loss from sport [1,2]. Despite excessive efforts in the area of hamstring injury prevention [3], hamstring strain injury rates increased from 12% to 24% during a 21-year UEFA Elite club injury surveillance, reporting a frequency of 1.7 injuries per 1000 h of total play, while match injury rates were 10 times higher than training (4.99/1000 h vs. 0.52/1000 h; RR 9.67, 95% CI 8.93 to 10.47) with a median time loss of 13 days [4], resulting in high cost for both the athletes and the teams [5].

A variety of intrinsic and extrinsic risk factors for HSI have been proposed. Non-modifiable factors include older age and previous injury, both of which have been consistently reported as strong predictors of future strain [6]. In fact, prior HSI has been identified as the single most robust risk factor, with relative risk estimates ranging from 2.3 to 6.1 in different cohorts [6], The mechanisms underlying this association are thought to include incomplete recovery of muscle architecture [7] persistent neuromuscular inhibition [8] and structural changes within the myotendinous junction [9]. Modifiable factors, such as hamstring strength [10], flexibility [11], fascicle length [7], endurance [12], and fatigue [13] have been the focus of many preseason screening batteries, but their predictive value has been inconsistent [14,15,16]. Several prospective studies have linked eccentric hamstring weakness to increased risk [17], whereas others have failed to demonstrate a significant association [18]. Residual weakness after an initial injury has been suggested to explain the high reinjury rates [4]. These discrepancies emphasize the multifactorial nature of HSI and the limitations of single-parameter screening.

While much of the literature has focused on the hamstrings themselves, attention has increasingly turned to adjacent muscle groups that may indirectly influence hamstring loading. The hip abductors have emerged as a plausible candidate, given their central role in lumbopelvic stability during running and cutting [19,20,21]. Functionally, the hip abductors stabilize the pelvis in the frontal plane and help maintain lower-limb alignment during stance [22,23]. Weakness in this group may allow excessive pelvic drop or anterior tilt, which alters hamstring length–tension relationships, particularly during the late swing phase of sprinting when hamstrings are lengthening while generating high force [24]. Poor abductor strength may also promote excessive knee valgus and increase iliotibial band tension, further disrupting energy transfer across the pelvis and trunk [25]. These alterations could increase mechanical demand on the hamstrings and predispose players to strain injuries [26,27]. Although the influence of hip abductors has been suggested in theoretical and biomechanical studies, prospective evidence linking abductor strength to HSI risk remains scarce.

Given the uncertainty around both direct and indirect strength measures, prediction models have been developed to integrate multiple risk factors [28]. Prediction requires multivariate approaches that can account for interactions among risk factors [29]. Traditional regression models have long been used to identify associations, but their translation into reliable prediction tools has been limited [30]. Several HSI prediction models have been published, often incorporating age, previous injury, and strength measures. Some reported apparently high discrimination (AUC > 0.80) [31] but systematic reviews and methodological critiques have highlighted important shortcomings: most models relied on small sample sizes, single baseline measures, and lacked external or temporal validation [32,33]. Many failed to assess calibration, used redundant predictors, and suffered from low events-per-variable ratios, which increase the risk of overfitting and optimism in reported performance [34,35,36]. Broader clinical research has shown that machine learning rarely outperforms logistic regression in such contexts [33]. In sports medicine, this has been reflected in inconsistent results: while some studies report promising classification accuracy [35,36] others find poor generalizability and instability of predictors when applied to new cohorts [37,38].

These limitations underline the importance of developing prediction models that are transparent, hypothesis-generating, and compliant with established reporting standards. Frameworks such as TRIPOD and PROBAST provide clear guidance for reporting, validation, and risk of bias assessment [39]. The recent TRIPOD-AI extension further emphasizes the need to address issues of overfitting, calibration, and predictor stability when applying machine learning or penalized regression approaches in biomedical research [39]. By situating new models within this framework, researchers can avoid overstating findings and contribute incremental knowledge rather than definitive screening tools.

Thus, while hip abductors are biomechanically plausible contributors to hamstring injury, their role as predictors has not been tested prospectively in large amateur cohorts. To date, no study has prospectively examined the role of hip abductor strength as a predictor of HSI in male amateur soccer players, who represent the largest playing population worldwide but remain underrepresented in research. Amateur players often lack the medical and conditioning support available in elite environments, which may increase their vulnerability to injury and alter the relevance of different risk factors.

The objectives of this study were as follows: (1) to develop and internally validate a multivariable prediction model for hamstring strain injuries in amateur soccer players, using preseason measures of isometric hip adduction, hip abduction, hip flexion, and hamstring strength; and relative ratios (2) to examine whether these strength variables, alongside previous injury history, contribute meaningfully to predicting injury risk; and (3) to evaluate the predictive accuracy of logistic regression and machine learning classifiers applied to the dataset. As a secondary aim, we also documented self-reported mechanisms of injury during follow-up, while acknowledging that these accounts were not objectively verified and should therefore be interpreted with caution. In line with TRIPOD + AI guidance, this represents a Category 2 study (model development without external validation). Given the small number of outcome events, the model should be considered exploratory and hypothesis-generating rather than confirmatory.

## 2. Methods

### 2.1. Study Design

This study followed an observational cohort design spanning of 30 weeks (August 2018 to April 2019) and aligning with the Strengthening the Reporting of Observational Studies in Epidemiology (STROBE) guidelines [40]. Our research group has previously conducted three studies using the same database-two on the epidemiology of musculoskeletal injuries [1,41,42] and one groin injury risk factors [42]. Building on this foundation, the present investigation focused on hamstring strain injury. Reporting follows the Transparent Reporting of a multivariable prediction model for Individual Prognosis or Diagnosis (TRIPOD) and TRIPOD-AI extensions [39]. A completed TRIPOD/TRIPOD-AI checklist with item-level page references is provided in Supplementary Materials S3. Methodological quality was assessed using the PROBAST and PROBAST-AI frameworks, which evaluate bias across four domains: participants, predictors, outcome, and analysis. The results of this assessment are reported in Supplementary Materials S4.

At baseline, all players underwent bilateral isometric strength testing of the hip adductors, abductors, flexors, and knee flexors. Players were prospectively followed throughout the competitive season, with injury incidence and exposure systematically recorded.

### 2.2. Participants

A priori sample size analysis was conducted using G*Power (latest ver. 3.1.9.7; Heinrich-Heine-Universität Düsseldorf, Düsseldorf, Germany) [43] for logistic regression with a small-to-moderate effect size (Cohen’s f^2^ = 0.05–0.25), α = 0.05, and power = 0.80. This yielded an indicative range of approximately 110–130 players, consistent with previous injury-prediction studies [44,45,46,47]. However, in prediction model development, methodological guidance emphasizes the number of outcome events per variable (EPV) and model optimism rather than total sample size as the key indicators of reliability [48]. Accordingly, the final model—with ten predictors and 21 injury events (EPV ≈ 2.1)—should be regarded as exploratory and hypothesis-generating, as performance estimates are likely unstable under these conditions. Eligible participants were male amateur soccer players ≥ 14 years, competing in the regional amateur soccer league, and free of musculoskeletal injury for ≥3 months before baseline. A total of 253 male players from 11 teams were screened during the 2018/19 off-season period (June to August). Of these, 176 players initially agreed to participate in the study, but 46 players were excluded due to non-compliance with the exposure limitations or inability to follow the data collection procedures. Ten players were excluded due to injury before the pre-season. The final cohort included 120 players. All trained in a standardized weekly micro cycle, consisting of four training sessions (Monday to Friday) designed to progressively build physical load, typically culminating in an official competitive match on the weekend (Saturday or Sunday). Written informed consent was obtained from all participants, and the study protocol was approved by the Aristotle University Ethical Committee (ERC-012/2019) in accordance with the Declaration of Helsinki.

### 2.3. Data Collection, Testing Protocol and Injury Registration

Baseline testing was performed in August 2018 at the medical facilities of the participating clubs. To standardize recovery status, all measurements were conducted on the 14th training day following a rest day between 17:00 and 18:30. Isometric strength of the hip adductors, hip abductors, hip flexors, and knee flexors was assessed bilaterally using a handheld dynamometer (KFORCE Muscle Controller, K-Invent, Montpellier, France). This device has demonstrated high intra-rater reliability and validity in musculoskeletal testing, with intraclass correlation coefficients reported above 0.80 for force and torque reliability and above 0.79 for validity [49,50]. Testing followed a standardized break-test protocol: each muscle group was tested twice with a 30-s rest interval, and the higher maximal voluntary contraction value was recorded. A two-minute rest period was provided between tests of different muscle groups to minimize fatigue. Muscle groups were tested in standardized positions: hip adduction and hip flexion in supine, hip abduction in side-lying, and knee flexion in prone with the knee at 15° of flexion. Hip extensor testing was not performed due to field-based logistical constraints. Accurate assessment of extensor force, particularly from the gluteus maximus, requires precise joint stabilization, torque alignment, and prolonged examiner involvement, which would have substantially increased testing duration in this large-cohort setting. Similar limitations in field-based hip extensor testing have been described previously [51]. From raw strength assessments, absolute values and selected ratios were derived, resulting in a total of 20 candidate predictors (Supplementary Materials S5). All strength variables were initially recorded in kilograms (kg) and normalised to body mass, yielding values expressed in Newton-metres per kilogram (Nm·kg^−1^). These continuous predictors were used in modelling. Two physiotherapists performed all testing: one served as lead examiner and the other assisted with participant positioning and stabilization. Both testers and all players were blinded to the recorded results for the duration of the study. Additional methodological detail and visual illustrations of the testing protocol are available in Supplementary Materials S1. Injuries were registered prospectively according to the FIFA/UEFA consensus definition [52]: any musculoskeletal complaint causing ≥1 missed training session or match. Data were collected with a standardized surveillance form (Supplementary Materials S2). Recorded items included date of injury, mechanism (sprinting, change of direction, or other), setting (training/match), reinjury status, and return-to-play duration.

### 2.4. Data Treatment and Statistical Analysis

All strength predictors were converted to Nm·kg^−1^ and then min–max scaled. Symmetrical uncertainty was used to rank predictors; the top 10 were retained. To mitigate collinearity, variance inflation factors (VIF) were computed; when VIF > 5, correlated predictors were clustered and the most clinically interpretable retained. All preprocessing (scaling, ranking, VIF checks) was nested strictly within training folds to prevent data leakage. The modelling outcome was a player-level binary indicator of hamstring strain injury during the season (injured vs. not injured). Data were split into training (70%; n = 83) and independent test (30%; n = 37) sets using stratification by injury status. Within the training set, four-fold cross-validation with nested resampling was used for hyperparameter tuning and optimism estimation. The primary model was logistic regression with elastic-net regularization; exploratory comparators included *k*-nearest neighbours and support vector machines under the same framework. No missing baseline data were present in the analysis dataset. Hyperparameters are shown in Supplementary Materials S6.

### 2.5. Development of the Logistic Regression Model

Logistic regression with an elastic-net penalty was fit to classify players as injured vs. not injured. The l1_ratio was tuned by nested four-fold cross-validation within the training set. All preprocessing (min–max scaling, feature ranking, and VIF-based pruning) was performed only within training folds. Models were implemented in PyCaret (Spyder IDE) with complementary analyses in Orange v3.4.0 and XLSTAT v2014.

### 2.6. Planned Model Evaluation Performance

Planned performance evaluation included discrimination (AUC with 95% CIs), calibration (slope and intercept, plus calibration plots), and classification metrics (accuracy, sensitivity, specificity, precision, F1). Confusion matrices for both training and test sets are provided in Supplementary Materials S7. Regression coefficients, odds ratios, and 95% CIs are presented in Supplementary Materials S8.

### 2.7. Planned Stability and Robustness Analyses

Model stability was examined using bootstrap resampling and permutation importance. Bootstrap resampling (200 iterations) refits the model on samples drawn with replacement from the training set and evaluated performance on the fixed test set. For permutation importance, each predictor was permuted 50 times in the test set while other variables were held constant; the mean change in AUC (ΔAUC) was recorded.

## 3. Results

### 3.1. Injury Incidence

Of the 120 participants (mean age 20.0 ± 6.9 years; BMI 22.5 ± 2.3 kg·m^−2^; height 1.77 ± 0.07 m; mass 70.7 ± 10.1 kg), 21 players sustained at least one hamstring strain injury during the season, for a total of 32 events (28% reinjuries). Most injuries occurred in the dominant limb (56.3%) and were attributed to sprinting activities (81.2%), based on player self-report without video or GPS confirmation. The majority were grade I strains, with a single grade II strain of the biceps femoris long head. Mean return-to-play duration was 9.3 ± 5.3 days (range 3–32). For prediction modelling, the outcome was analyzed at the player level (injured vs. not injured).

### 3.2. Model Performance

**Figure 1:**
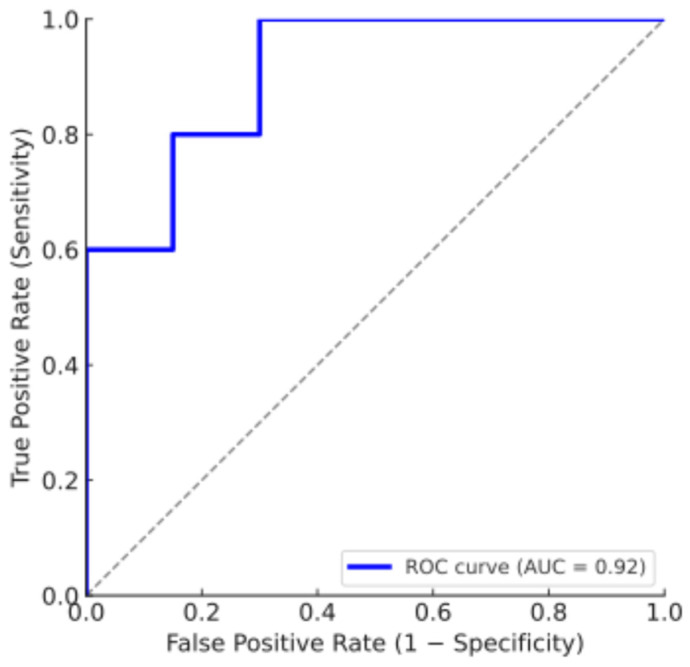
Receiver operating characteristic (ROC) curve for the logistic regression model (AUC = 0.92). The blue line represents the model’s discriminative performance, while the gray dashed diagonal line indicates the no-skill reference (AUC = 0.50), corresponding to random classification.

**Figure 2:**
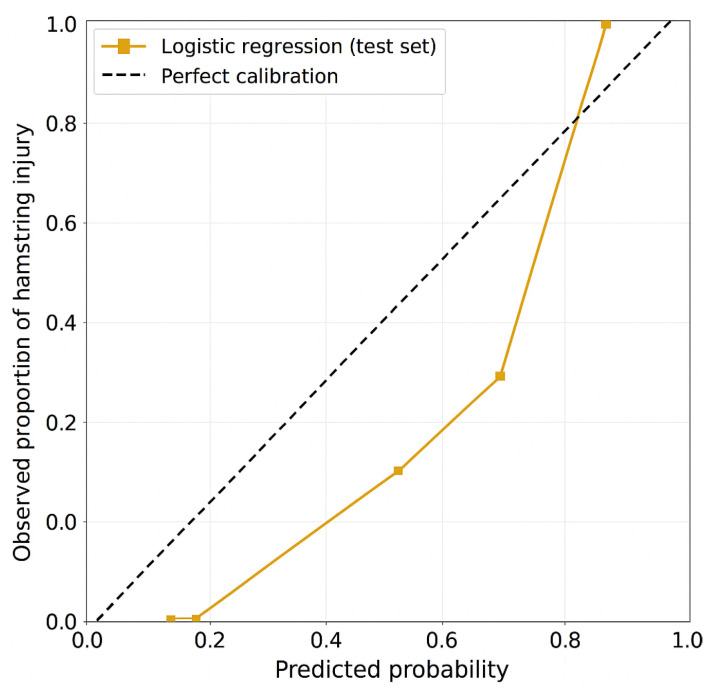
Calibration plot for the logistic regression model on the test set. The diagonal line represents perfect agreement between predicted and observed probabilities of hamstring injury. The model showed a calibration slope of 0.85 and an intercept of −0.12, indicating mild overfitting and a slight underestimation of true injury risk. Overall, the model demonstrated reasonable alignment between predicted and observed probabilities, but the calibration performance remains insufficient for clinical application.

On the training set (n = 83; 16 injured, 67 uninjured), the logistic regression with an elastic-net penalty model achieved an accuracy of 76.4% (64/83 correctly classified), with sensitivity 81.3% (13/16 injured) and specificity 76.1% (51/67 uninjured). On the independent test set (n = 37; 5 injured, 32 uninjured), accuracy declined to 64.9% (24/37 correct), with sensitivity 60% (3/5 injured) and specificity 65.6% (21/32 uninjured). The not-injured class comprised TN + FP = 21 + 11 = 32 players. Discrimination was modest, with AUC = 0.68 (95% CI 0.52–0.84) (Figure 1). Calibration analysis indicated mild overfitting with slight overestimation of risk (slope 0.85, intercept −0.12) (Figure 2).

To complement these split-sample results, Table 1 shows cross-validation performance metrics, with mean accuracy 69.9% (±3.8), AUC 0.79 (±0.06), sensitivity 0.70 (±0.07), precision 0.90 (±0.05), and F1 score 0.79 (±0.04). These results confirm moderate discrimination but limited sensitivity, reducing clinical utility.

### 3.3. Predictor Importance

**Figure 3:**
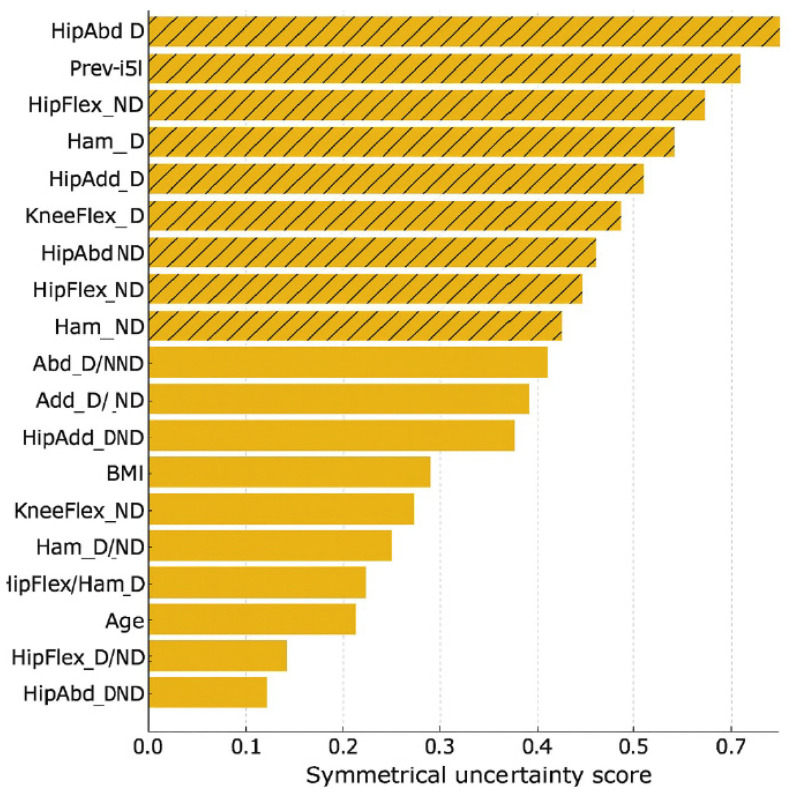
Variable ranking based on symmetrical uncertainty. Relative predictor importance among the 20 candidate variables. Ten predictors (hatched bars) were retained for model development, with dominant-leg hip abduction strength (HipAbd_D) emerging as the top-ranked feature. These rankings suggest a possible role for hip abduction strength in hamstring-injury risk profiling, but they should be interpreted with caution given the small number of outcome events (EPV = 2.1) and exploratory nature of the model.

Feature ranking identified ten of the original twenty variables as influential (Figure 3). In the final multivariable logistic regression with an elastic-net penalty model, only dominant-leg hip abduction strength remained statistically significant (OR 0.82, 95% CI 0.70–0.96, *p* = 0.016), suggesting a protective association. Age, BMI, previous injury, and hamstring strength were not significant predictors (Table 2). The Hosmer–Lemeshow test (*p* = 0.98) and Nagelkerke R^2^ (0.29) are reported for completeness but are interpreted cautiously, given the low events-per-variable ratio (2.1).

### 3.4. Stability Analyses

**Figure 4:**
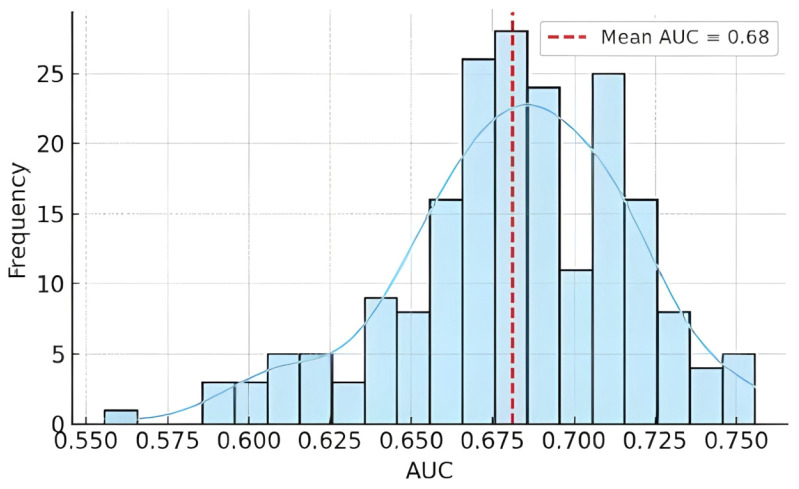
Distribution of AUC values across 200 bootstrap resampling iterations. The histogram depicts the frequency of AUC values, while the blue curve represents the smoothed kernel density estimate of their distribution. The red dashed line marks the mean AUC (0.68), indicating moderate discriminative ability of the model. The broad spread of AUC values illustrates considerable variability and highlights the instability of performance estimates under the low events-per-variable ratio, consistent with the exploratory purpose of this analysis.

**Figure 5:**
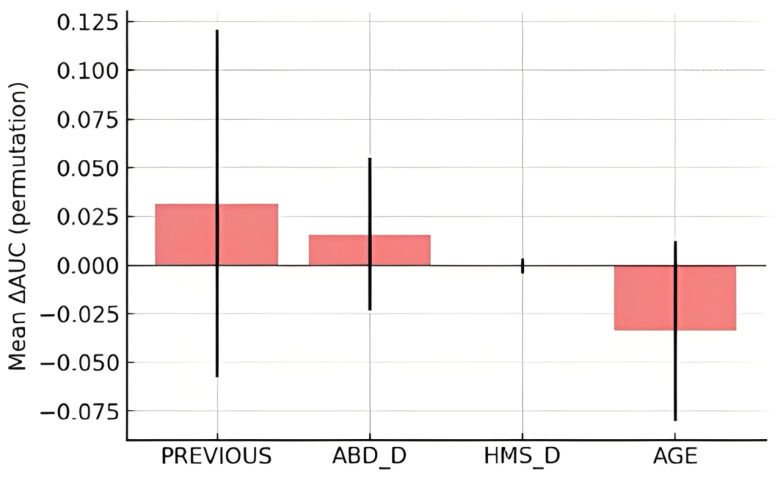
Permutation importance of key predictors in the test set. Bars show the mean change in AUC (ΔAUC) across 50 permutations, with error bars representing standard deviation. Previous hamstring injury produced the largest decrease in model performance, confirming it as the dominant predictive factor. Dominant-leg hip abduction strength (ABD_D) contributed modestly, whereas hamstring strength (HMS_D) and age had negligible or inconsistent effects. The wide confidence ranges highlight substantial uncertainty due to the limited sample and low events-per-variable ratio, reinforcing the exploratory nature of these results.

On the independent test set, the baseline logistic regression model achieved an AUC of 0.68 (95% CI 0.52–0.84). Bootstrap resampling (n = 200) yielded a mean AUC of 0.681 (SD 0.036; 2.5th–97.5th percentiles 0.600–0.745), indicating modest and uncertain discrimination consistent with the small number of events (Figure 4). Permutation importance confirmed previous hamstring injury as the strongest contributor to model performance (mean ΔAUC −0.032 ± 0.089), followed by dominant-leg hip abduction strength (ΔAUC −0.016 ± 0.039). Hamstring isometric strength exerted negligible influence (ΔAUC ≈ 0), and age produced no meaningful reduction (ΔAUC ≈ +0.034, consistent with noise) (Figure 5). These results support treating hip abduction as a candidate signal and reaffirm the established contribution of previous injury, while underscoring instability at EPV ≈ 2.1. Full distributions from bootstrap resampling and detailed permutation importance scores are provided in Supplementary Materials S9.

## 4. Discussion

The primary findings of this investigation revealed that previous hamstring injury was the strongest predictor of subsequent hamstring strain injury (HSI), even though it did not reach conventional significance in the multivariable model, reflecting limited power. This is consistent with extensive literature identifying prior injury as the most robust risk factor in soccer [6]. In addition, reduced dominant-leg hip abduction strength emerged as a candidate signal, although bootstrap resampling and permutation importance indicated that this association was unstable at the current events-per-variable ratio (≈2.1). By contrast, isometric hamstring strength and age did not demonstrate predictive value in this cohort. Although not a primary study objective, descriptive injury surveillance confirmed that sprinting was the most frequently reported injury mechanism, aligning with prior reports that high-speed running is the predominant context for HSIs in soccer [53,54]. Most injuries were low grade, with rapid return-to-play, but details on anatomical site and mechanism could not be verified due to reliance on self-report.

This study is the first to explore dominant-side hip abduction strength as a potential contributor to HSI susceptibility in amateur soccer. The hip abductors play a role in frontal-plane stability and lumbopelvic control, and weakness in this group may compromise energy transfer and increase mechanical demand on the hamstrings [8,22]. However, the instability of our abductor signal highlights the risk of over-interpretation. A clearer mechanistic understanding will require future studies incorporating motion analysis and electromyography to evaluate lumbopelvic control during high-speed running. Although the abductor signal in our model was unstable, the potential link between dominant-side hip abduction weakness and HSI risk is biologically plausible. Prior research has focused primarily on intramuscular risk factors such as eccentric hamstring strength, fascicle length, endurance, and flexibility, while less attention has been given to the role of adjacent anatomical and functional structures, particularly the lumbar spine and pelvic stabilizers [20,21]. The hip abductors contribute to frontal-plane stability and energy transfer across the lumbopelvic region. Deficits in this group may compromise trunk and pelvic neuromuscular control, mechanisms that have been linked to lower-limb injury risk [8,19]. For instance, hip abductor weakness may promote excessive knee valgus, thereby increasing tension on the iliotibial band during the early stance phase of running, particularly when deceleration occurs to absorb ground reaction forces [19]. Although our study did not directly measure kinematic variables or muscle activation, our findings are consistent with existing theories suggesting that improved lumbopelvic control may contribute to reduced hamstring injury risk [8]. The relationship between hamstring strength and HSI risk remains debated. While some prospective studies suggest that eccentric hamstring weakness increases risk, others report limited predictive value [44,45]. Our finding that isometric hamstring strength was not significant aligns with the latter, but methodological variability in strength testing and outcome definition likely contribute to discrepancies across studies [55]. Furthermore, our finding that age was not significantly associated with injury risk must be interpreted in light of the sample characteristics: the relatively young mean age of participants (20.0 ± 6.96 years) may have limited the ability to detect an age-related effect, which is more consistently observed in older or elite-level cohorts.

### 4.1. The Predictive Value of Machine Learning Algorithms

Our model achieved modest discrimination, with a test-set AUC of 0.68 (95% CI 0.52–0.84). This level of performance is comparable to other small-sample studies of preseason screening, where AUC values typically range between 0.60 and 0.75 and rarely exceed 0.80 [22,26,32,33]. While Ayala et al. [31] reported a higher AUC of 0.83 when including hip isometric strength in their model, their approach was subject to similar limitations of small event counts, baseline-only measures, and lack of external validation. The consistent challenge across studies is that models often show promising apparent discrimination but fail to generalize when tested in independent samples [56]. Importantly, discrimination alone provides an incomplete picture. Calibration—the agreement between predicted and observed risks—is equally critical for clinical utility [57]. Our calibration analysis indicated mild overfitting (slope 0.85, intercept −0.12), reinforcing the need for shrinkage and external validation. These issues are common: most sports injury prediction studies are underpowered, with low events-per-variable ratios, which inflates optimism and limits reproducibility [58]. The appeal of machine learning lies in its ability to accommodate complex, non-linear interactions among multiple risk factors. However, systematic reviews consistently show that ML rarely outperforms logistic regression when sample sizes are modest and predictors are limited [59]. Our findings mirror this evidence: logistic regression, combined with internal resampling, provided performance similar to what has been reported for more complex models, with the added benefit of interpretability and transparency. Therefore, while ML methods hold promise for multifactorial risk profiling, their predictive value in practice will depend on large, multicenter datasets, richer longitudinal features (e.g., training load, fatigue indices, neuromuscular control), and rigorous validation strategies. In the current dataset, the model is best interpreted as exploratory, hypothesis-generating, and not ready for clinical deployment.

### 4.2. Strengths and Limitations

A key strength of this study was the prospective cohort design, with preseason baseline testing and systematic surveillance across a full competitive season. Monitoring 120 players over 30 weeks ensured consistent exposure tracking in an amateur context. Strength testing followed a standardized and reliable protocol, performed by a single experienced physiotherapist, which minimized inter-rater variability. Although belt fixation was not feasible in the field, examiner-stabilized provide acceptable reliability [30]. Internal validity of the assessments can therefore be considered high. Methodological transparency was another strength. The study adhered to STROBE and incorporated key TRIPOD-AI elements, including clear definitions of predictors, outcomes, and modelling strategy. Beyond conventional outputs, bootstrap resampling and permutation importance analyses were applied to explore model stability, and a PROBAST-AI identified high risk of bias overall, particularly in the analysis domain due to low EPV and lack of external validation. Several limitations must be acknowledged. The number of events was small (21 HSIs), producing a low events-per-variable ratio (~2.1) and increasing risk of overfitting. Under such conditions, predictive performance indices such as AUC and calibration should be interpreted with extreme caution, as they are likely unstable and may not reflect true model performance. External or temporally separated validation was not performed, so findings should be interpreted as hypothesis-generating. Injury mechanisms were self-reported and could not be verified by imaging or GPS, limiting precision. The predictor set was restricted to isometric strength and a few contextual variables, excluding neuromuscular or load-related measures. Finally, external validity is limited: the cohort consisted of young male amateur players, and results may not generalize to elite, female, or older populations.

### 4.3. Future Directions

Future research on hamstring strain injury prediction should prioritize larger, multicenter cohorts to increase the number of outcome events and improve reliability of modelling. Such studies should follow methodological guidance on prediction model development, ensuring adequate events-per-variable ratios, use of shrinkage, and thorough calibration. External and temporally separated validation datasets are essential to establish generalizability, since even apparently strong discrimination can reflect overfitting in small samples. The predictor set also requires expansion beyond isometric strength. Incorporating eccentric hamstring measures, hip extensor and rotator strength, and dynamic neuromuscular assessments would provide a more complete biomechanical profile. Longitudinal monitoring of workload (e.g., GPS-derived load, training-to-match ratios, acute: chronic workload), along with markers of fatigue, recovery, and contextual factors such as sleep, wellness, or playing position, may better capture the dynamic nature of injury risk. Advanced measurement tools could further clarify mechanistic pathways. Motion capture or wearable sensors can quantify sprinting mechanics, lumbopelvic control, and deceleration strategies, while electromyography may reveal deficits in coordination or activation patterns. These approaches could determine whether the observed signal from hip abductor weakness reflects a true causal role or a spurious finding related to sample limitations. From an analytical perspective, future studies should embed rigorous resampling and validation strategies, including bootstrap, permutation importance, and nested cross-validation. Where datasets are sufficiently large, penalized regression, ensemble methods, or neural networks may be appropriate. However, systematic reviews show that machine learning does not consistently outperform logistic regression in modest datasets [59]. Emphasis should therefore remain on methodological transparency, calibration, and reproducibility rather than algorithm novelty. Finally, translation into practice requires impact evaluation. Beyond development and validation, research should test whether integrating predictors such as hip abduction strength into screening protocols influences clinical decision-making, reduces injury incidence, or improves rehabilitation outcomes. Such impact studies are the final step in the TRIPOD framework but are largely absent in sports injury research. A PROBAST-AI assessment indicated overall high risk of bias, mainly due to the low number of outcome events, the low events-per-variable ratio, and the absence of external validation. Risk was lower in the domains of participants and predictors, given the prospective design and standardized testing, but concerns were high in the analysis domain because of potential overfitting and instability. This reinforces the interpretation of our findings as exploratory and hypothesis-generating.

### 4.4. Clinical Relevance

The present findings should be interpreted within the specific context of young male amateur soccer players and should not be generalized to elite, professional, or female populations, where physical demands, neuromuscular profiles, and injury mechanisms differ considerably. Currently, a history of previous hamstring injury remains the most reliable screening variable and should continue to guide clinical risk assessment. While hip abductor strength emerged as a potentially meaningful factor, the evidence is still exploratory and lacks sufficient robustness for clinical decision-making. Nevertheless, these results may inform the design of future research and screening frameworks by highlighting the need to (1) include multi-joint and kinetic chain–based assessments rather than focusing solely on hamstring strength, (2) explore the predictive contribution of hip abductor strength in combination with other neuromuscular or workload variables, and (3) validate such models across diverse playing levels, sexes, and competition demands. Until larger, longitudinal, and externally validated studies confirm these associations, the current findings should be regarded as hypothesis-generating and not yet suitable for direct clinical application.

## 5. Conclusions

This prospective cohort study identified previous hamstring injury as the most consistent predictor of subsequent HSI, consistent with established evidence. Reduced dominant-leg hip abduction strength also emerged as a candidate risk factor, but its instability under bootstrap and permutation analyses indicates that this association should be interpreted cautiously. Neither isometric hamstring strength nor age were significant predictors in this young amateur cohort. Model performance was modest, calibration indicated mild overfitting, and PROBAST-AI assessment classified the analysis domain as high risk for bias due to the low events-per-variable ratio and absence of external validation. The study’s reliance on isometric strength variables represents an important limitation. Future research should incorporate eccentric strength testing, dynamic neuromuscular assessments, and workload-related measures, which are likely to yield more accurate and ecologically valid predictors of injury risk. Collectively, these findings highlight both the limited predictive value of isolated preseason strength screening and the potential relevance of lumbopelvic and hip function for developing more comprehensive injury prediction frameworks. Larger, multicenter cohorts with longitudinal, multifactorial datasets and rigorous external validation are required before such models can inform clinical decision-making. Until then, the current results should be viewed as hypothesis-generating rather than clinically actionable.

## Figures and Tables

**Table 1:** Cross-validation performance of the logistic regression model for predicting hamstring strain injury risk. Metrics are reported as mean ± SD across cross-validation folds.

Metric	Mean	SD	Description
Accuracy (%)	69.9	3.8	Proportion of all correctly classified cases
AUC	0.792	0.064	Area under the ROC curve
Sensitivity (Recall)	0.700	0.073	True positive rate (injured correctly class).
Precision	0.901	0.053	Positive predictive value
F1 Score	0.787	0.040	Harmonic mean of precision and recall

**Table 2:** Final multivariable logistic regression model for hamstring strain injury (player-level outcome) Note: OR = odds ratio; CI = confidence interval; D = dominant leg; ND = non-dominant leg. Full model specification is provided in Supplementary Materials S8. * Statistically significant at *p* < 0.05.

Predictor	β (SE)	OR	95% CI	*p*-Value
Intercept	0.963 (4.334)	2.62	0.10–12.80	0.824
Age (years)	−0.012 (0.050)	0.99	0.90–1.09	0.808
BMI (kg/m^2^)	0.034 (0.136)	1.03	0.79–1.35	0.805
Previous hamstring injury	−1.283 (0.805)	0.28	0.06–1.34	0.111
Hip abduction (dominant leg)	−0.200 (0.083)	0.82	0.70–0.96	0.016 *
Hip flexion (non-dominant leg)	0.108 (0.067)	1.11	0.98–1.27	0.109
Hip adduction ratio (D/ND)	0.939 (1.520)	2.56	0.13–50.27	0.536
Hip abduction ratio (D/ND)	−0.414 (1.121)	0.66	0.07–5.95	0.712
Hamstring ratio (D/ND)	−0.346 (1.541)	0.71	0.04–14.51	0.822
Hip flexion ratio (D/ND)	2.304 (2.251)	10.02	0.12–826.22	0.306
Hip flexion/hamstring ratio (dominant leg)	−0.621 (0.947)	0.54	0.08–3.44	0.512

## Data Availability

The data presented in this study are available upon request from the corresponding author.

## Supplementary Materials

The following supporting information can be downloaded at https://www.mdpi.com/article/10.3390/muscles4040050/s1. Supplementary Material S1: Testing protocol, Supplementary Material S2: Injury data registration, Supplementary Material S3: TRIPOD-AI Checklist, Supplementary Material S4: PROBAST-AI Risk of Bias Assessment (Hamstring Prediction Model), Supplementary Material S5: Candidate predictors and definitions, Supplementary Material S6: Model Specifications and Hyperparameters, Supplementary Material S7: Confusion Matrices for logistic regression model, Supplementary Material S8: Final logistic regression model coefficients (player-level outcome), Supplementary Material S9: Stability analyses: bootstrap resampling and permutation importance.
