# Supplementary material for: Hamstring Strain Injury Risk in Soccer: An Exploratory, Hypothesis-Generating Prediction Model
Source: Muscles. 2025 Nov 4;4(4):50. doi: 10.3390/muscles4040050 (PMC12641714; doi:10.3390/muscles4040050)
Supplement: Supplementary file 1 [file muscles-04-00050-s001.zip › muscles-3908730-supplementary.pdf]

Supplementary Material S1

Testing protocol

In our study, we observed that not all teams commenced their pre-season program simultaneously. To ensure consistency, we scheduled the measurement day precisely two weeks after the initiation of the training session, which occurred in August 2018. This day was chosen to be the 14th training day, following a day of recovery, and took place between the hours of 17:00 and 18:30. Although the number of players varied among the teams, it is important to note that all players from each team were measured during this session. The study collected the anthropometric characteristics of the players, including age, stature, and body mass, and calculated their body mass index (BMI). The participants' preferred legs and medical history were additionally recorded. All players were assessed once, during the begging of the pre-season, and were followed for 30 weeks until the end of the season, including play-off matches.

The medical office of each soccer club was used for testing procedures. The set-up included a physiotherapy table, a tablet, and a portable HDD (KFORCE Muscle Controller, K-Invent, Biomecanique, Montpellier, France). The medical office of each soccer club was used for testing procedures. All Participants performed a warm-up consisting in 5-min of jogging in light intensity and an 8-min protocol of standardized static stretching exercises in leg and trunk muscles(Cejudo et al. 2015).

Two physiotherapists, a tester [redacted] and an assistant [redacted] were present during the testing sessions. The tester, (A.K.) with extensive experience using the HHD, performed all the testing. The assistant (T.B.) registered all hip strength measurements and assisted in patient measurement fixations. The tester was blinded to the hip muscle strength of participants.

The tests were conducted in a predetermined sequence. To ensure correct action, the investigator's hand and the other hand were placed against HHD. The players were instructed to stabilize themselves by holding onto the sides of the table. Resistance was applied in a fixed position, and the participant exerted a 3-second isometric maximum voluntary contraction (MVC) against the dynamometer and the examiner. Each test was performed bilaterally, starting with the right limb, and administered 2 times with a 30-second resting period in between. The highest value of the 2 valid MVCs was used in the analysis. Participants were asked to resist the applied force (break test) (der Ploeg, G H Oosterhuis, and der Ploeg J G H Oosterhuis 1991). To avoid potential fatigue, a 2-minute rest period was implemented between each of the four tests (ref). The highest score achieved in each test was utilized for subsequent data analysis and treatment. The supine position was used for testing isometric hip adduction (ADD) strength (image 1). The hips were slightly abducted to fit the angle of the tester's elbows as previously described (Nielsen et al. 2022). The isometric hip abduction (ABD) strength was tested in side-lying position as previously described (Thorborg et al. 2011), (2011), (image2.) The isometric knee flexion (HMS) strength was tested in prone position with the knee to 15° as described by Reurink and colleagues(Reurink et al. 2016) (2016), (image 3). The supine position was applied for testing isometric hip flexion (HFL) strength as described by Thorborg and colleagues(Thorborg et al. 2010), (2010), (Image 4)

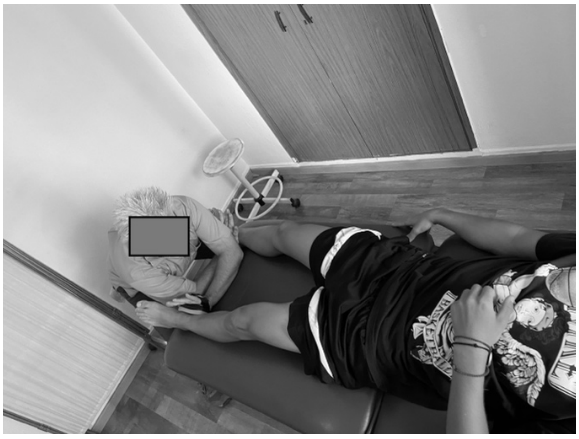

Figure S1. Isometric hip adduction strength test (Nielsen et al., 2022)

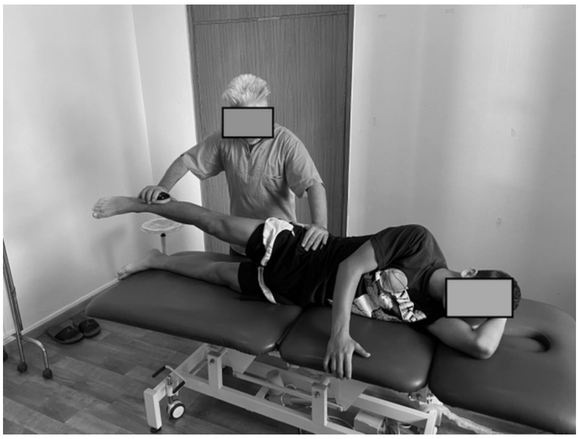

Figure S2: isometric hip abduction (ABD) strength test (Thorborg et al., 2011)

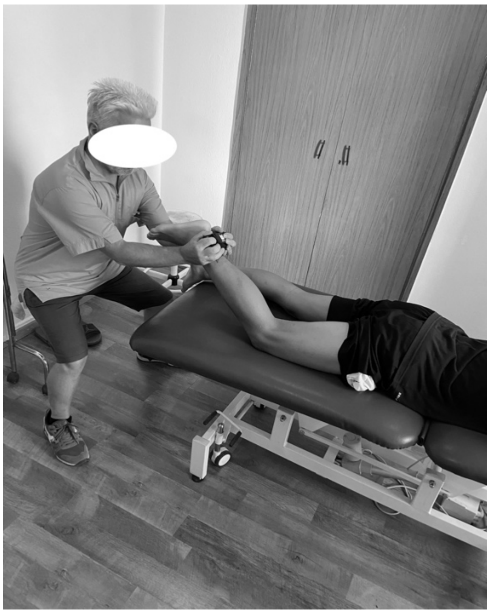

Figure S3: Isometric knee flexors strength test in lengthening position (Reurink et al., 2016)

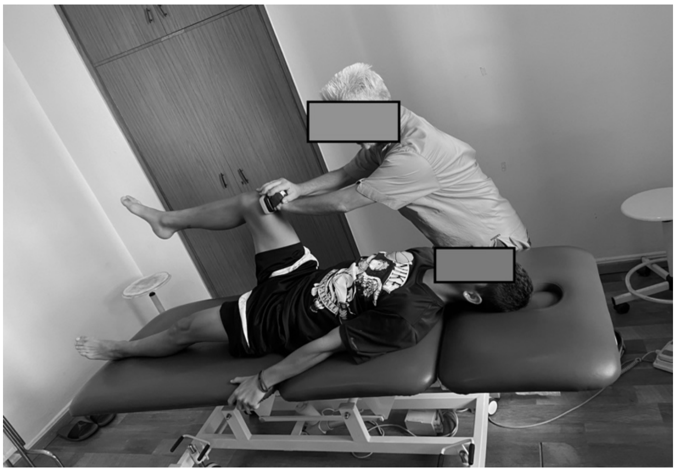

Figure S4. Isometric hip flexors strength test (Thorborg et al., 2010)

Supplementary Material S2. Injury data registration

Plar	Age	Position	Height	Weight	BMI*	Dominant	Previous Injury	Date of injury	injury	Mechanism of injury	Game or Training	Re-injury	RTP days	Use of imagine
player 1	32	Defence winger	1.68	62	22.0	0	1	14th sep 18	1st Grade injury R HIS	sprinting	T	NO	5	NO
player 2	30	Defence winger	1.76	83	26.8	0	1	9th sep 18	1st Grade injury L HIS	sprinting	T	NO	7	NO
player 2	30	Defence winger	1.76	83	26.8	0	1	20th Oct 18	1st Grade injury L HIS	Sprinting	G	yes	14	yen
player 1	32	Defence winger	1.68	62	22.0	0	1	20th Oct 18	1st Grade injury L HIS	sprinting	G	NO	5	NO
player 3	28	Attacking Winger	1.74	71	23.5	0	1	28th Oct 18	1st Grade injury R HIS	sprinting	G	NO	8	NO
player 1	32	Defence winger	1.68	62	22.0	0	1	15th Dec 18	1st Grade injury R HIS	Sprinting	T	yes	11	NO
player 4	20	Defence winger	1.76	83	26.8	0	1	16th Jan 19	1st Grade injury R HIS	Sprinting	T	NO	6	NO
player 1	32	Defence winger	1.68	62	22.0	0	1	24th Jan 19	1st Grade injury R HIS	kicking	T	yes	17	yes
player 5	28	Defece Middle	1.71	73	25.0	0	1	6th Feb 19	1st Grade injury L HIS	Sprinting	G	NO	3	NO
player 5	28	Defece Middle	1.71	73	25.0	0	1	17th Feb 19	1st Grade injury L HIS	sprinting	T	yes	9	NO
player 2	30	Defence winger	1.76	83	26.8	0	1	6th Mar 19	1st Grade injury L HIS	Sprinting	G	yes	12	NO
player 2	30	Defence winger	1.76	83	26.8	0	1	24th Apr 19	1st Grade injury R HSI	Sprinting	G	NO	6	NO
player 6	15	forward	1.84	76	22.4	0	1	14th Oct 18	1st Grade injury L HIS	sprinting	G	NO	8	NO
player 6	15	forward	1.84	76	22.4	0	1	4th Nov 18	1st Grade injury R HSI	sprinting	G	NO	12	NO
player 6	15	forward	1.84	76	22.4	0	1	17th Mar 19	1st Grade injury R HSI	sprinting	G	yes	19	yes
player 7	17	Attacking Winger	1.76	65	21.0	0	0	3rd Sep 18	1st Grade injury L HIS	sprinting	T	NO	8	NO
player 8	21	Attacking Winger	1.80	70	21.6	0	1	14th Oct 18	1st Grade injury L HIS	sprinting	G	NO	6	NO
player 8	21	Attacking Winger	1.80	70	21.6	0	1	10th Feb 19	1st Grade injury L HIS	sprinting	G	yes	9	NO
player 9	33	Attacking Winger	1.72	77	26.0	0	1	13th Feb 19	1st Grade injury L HIS	sprinting	T	NO	10	NO
player 10	40	Central defender	1.80	85	26.2	0	1	2nd Oct 18	1st Grade injury L HIS	sprinting	T	NO	7	NO
player 11	37	Attacking Midfielder	1.70	60	20.8	0	1	5th Oct 18	1st Grade injury R HIS	sprinting	T	NO	6	NO
player 12	33	Center Middfielder	1.80	78	24.1	0	1	17th Oct 18	1st Grade injury L HIS	sprinting	G	NO	8	NO
player 13	26	Attacking Middle center	1.72	80	27.0	0	1	13th Nov 18	1st Grade injury R HIS	sprinting	G	yes	11	NO

player 13	26	Attacking Middle center	1.72	80	27.0	0	1	15th Jan 19	1st Grade injury R HIS	Acceleration	T	yes	9	NO
player 14	16	Defencive Midfielder	1.75	70	22.9	0	1	10th Mar 19	1st Grade injury R HIS	sprinting	T	NO	7	NO
player 15	28	Goalkeaper	1.85	85	24.8	0	1	20th Nov 18	1st Grade injury L HIS	Acceleration	T	NO	9	NO
player 16	15	Attacking Winger	1.80	65	20.1	0	0	13st Oct 18	1st Grade injury R HSI	deceleration	G	NO	7	NO
player 17	22	Center Middfielder	1.68	65	23.0	0	1	10th Nov 18	2nd Grade R BFLh	sprinting	G	NO	32	yes
player 18	15	Defence winger	1.72	78	26.4	0	0	24th Jan 19	1st Grade injury R HIS	over stretching	G	NO	11	NO
player 19	15	Center Middfielder	1.76	58	18.7	0	1	4th Feb 19	1st Grade injury R HIS	Acceleration	T	NO	7	NO
player 20	31	Attacking Winger	1.76	64	20.7	0	1	18th Nov 18	1st Grade injury L HIS	deceleration	G	NO	6	NO
player 21	36	Central Middfielder	1.82	85	25.7	0	1	22th Nov 18	1st Grade injury L HIS	sprinting	T	NO	5	NO

Dominant limb: 0= Right, 1=Left

Previous injury: 0=No previous, 1= Previous

Game or Training : T= Training, G=Game

Use of imagine : Yes = MRI and/or MSK US

<u>Injury data registration</u>

A hamstring injury was described as "an injury to the posterior thigh resulting from playing football that caused a player to be unable to fully participate in future training or match play"[17]. Hamstring injuries reported as spasm/cramping by the player (in the absence of tissue damage), were included as injuries. Injury data were collected every week, verified by the first author [redacted], and subsequently collected. During the competitive season, all injuries were diagnosed and recorded by the medical staff of the football team or the medical staff of the local hospital following the recommendations adopted by FIFA[17]. The time devoted to training and match play was individually obtained by the team staff to calculate the time of exposure.

Suplementary Material S3. TRIPOD-AI Checklist

Title / Abstract	Identify study as developing a prediction model, specify population, outcome, and analysis type.	Title: “Hamstring strain injury risk in soccer: an exploratory, hypothesis-generating prediction model.” Abstract clearly states development of logistic regression model with internal validation only, in amateur male soccer players.
Introduction	Background and rationale for developing the model; reference to existing models.	Introduction discusses HSI prevalence, known risk factors, limitations of existing models, and justification for including hip abductors. Prior models (Ayala et al.) are cited.
Objectives	Clearly state whether the study is model development, validation, or both.	Objectives section specifies development and internal validation only; classified as TRIPOD Category 2 (development without external validation).
Source of Data	Study design, setting, and eligibility criteria.	Prospective cohort, 120 amateur male soccer players from 11 clubs, one competitive season. Eligibility: >14 years, regional league, injury-free at baseline.
Outcome	Define the outcome, blinding of outcome assessment.	Primary outcome: time-loss hamstring strain injury. Diagnosis based on medical reports and player self-report. No imaging/video confirmation. Modelled as binary (injured vs not).
Predictors	Define all candidate predictors, measurement methods, blinding.	Preseason isometric strength testing of hip adductors, abductors, flexors, hamstrings (bilateral). Predictors recorded with handheld dynamometer, normalised to body mass. Blinded testers. Previous injury history included.
Sample Size	Explain how study size was determined, including events-per-variable considerations.	G*Power used initially; revised to acknowledge 21 events with ~10 predictors (EPV ≈ 2.1), below recommended thresholds. Overfitting risk noted.
Missing Data	Describe handling of missing data.	No imputation performed; players with incomplete baseline testing excluded.
Statistical Analysis	Predictor handling, model type, validation, performance metrics.	Logistic regression with elastic-net penalisation. Predictors normalised and variance inflation checked. 70:30 train/test split with 4-fold cross-validation. Performance: accuracy, AUC, calibration slope/intercept, confusion matrix. Stability checked with bootstrap resampling (200 samples) and permutation importance.

**Risk Groups**	Define if risk groups were created.	Not created; probability estimates reported continuously.
**Development vs Validation**	Internal validation strategy, external validation.	Internal only: 4-fold CV + independent test set. No external validation.
**Performance**	Report discrimination, calibration, classification performance with CIs.	AUC 0.69 (95% CI 0.52–0.84 test set), calibration slope 0.85, intercept −0.12, sensitivity 60%, specificity 65.6%. Bootstrap distribution reported (AUC mean 0.68, 2.5–97.5% 0.60–0.75).
**Model Presentation**	Full model coefficients, intercept, or online calculator.	Regression coefficients, ORs, and 95% CIs presented in Table 2 and Supplementary Table S4.
**Limitations**	Discuss study limitations.	Low EPV, small sample, binary outcome collapsing reinjuries, no external validation, self-reported mechanisms.
**Interpretation**	Compare results with other studies, implications.	Discussion compares with Ayala et al. (2019) and broader evidence. Hip abductor finding discussed as candidate, unstable signal.
**Funding**	Sources and role of funders.	Reported in manuscript.

Supplementary Material S4 PROBAST-AI Risk of Bias Assessment (Hamstring Prediction Model)

Domain	Signaling Questions	Judgement	Comments
**Participants**	Was the study population representative of the target population?	Low risk	120 amateur male soccer players, 11 teams. Reasonably representative for regional-level male amateur soccer. Applicability limited to this group.
**Predictors**	Were predictors defined and measured consistently and blinded to outcome?	Low risk	Standardised isometric testing of hip and hamstring groups. Same device and examiner. Players and testers blinded to injury outcomes. Predictors restricted to preseason only.
**Outcome**	Was outcome definition appropriate, objective, and consistent?	High risk	Injuries prospectively recorded but based on medical report + player self-report, without video/GPS confirmation. Reinjuries collapsed into binary outcome (injured vs not).
**Analysis**	Adequate EPV, penalisation, calibration?	High risk	21 injured players vs ~10 predictors → EPV ≈ 2.1, far below recommended thresholds. Logistic regression with elastic-net used, but overfitting risk very high. Calibration and bootstrap/permutation stability analyses were added post-review, but no external validation.

Domain	Signaling Questions	Judgement	Comments
**Overall Risk of Bias**	Considering all domains	High risk	Small number of events, low EPV, collapsed outcomes, no external validation.
**Applicability**	Relevance to practice	Moderate	Findings limited to young male amateurs. Cannot be generalised to elite, female, or other populations. Predictors were limited (no eccentric hamstrings, no GPS load, no biomechanical data)

Supplementary Material S5. Candidate predictors and definitions

Predictor	Type	Unit	Definition
Age	Contextual	years	Chronological age at preseason testing
BMI	Contextual	$kg·m^{-2}$	Body mass index (body mass/height^2)
Previous injury	Contextual (binary)	yes/no	Self-reported hamstring injury within last season
Hip adduction strength (D)	Absolute	$Nm·kg^{-1}$ (normalised)	Maximal isometric hip adduction, dominant leg
Hip adduction strength (ND)	Absolute	$Nm·kg^{-1}$ (normalised)	Maximal isometric hip adduction, non-dominant leg
Hip abduction strength (D)	Absolute	$Nm·kg^{-1}$ (normalised)	Maximal isometric hip abduction, dominant leg
Hip abduction strength (ND)	Absolute	$Nm·kg^{-1}$ (normalised)	Maximal isometric hip abduction, non-dominant leg
Hip flexion strength (D)	Absolute	$Nm·kg^{-1}$ (normalised)	Maximal isometric hip flexion, dominant leg
Hip flexion strength (ND)	Absolute	$Nm·kg^{-1}$ (normalised)	Maximal isometric hip flexion, non-dominant leg
Hamstring strength (D)	Absolute	$Nm·kg^{-1}$ (normalised)	Maximal isometric knee flexion, dominant leg
Hamstring strength (ND)	Absolute	$Nm·kg^{-1}$ (normalised)	Maximal isometric knee flexion, non-dominant leg
Hip adduction ratio (D/ND)	Ratio	—	Ratio of dominant to non-dominant hip adduction strength
Hip abduction ratio (D/ND)	Ratio	—	Ratio of dominant to non-dominant hip abduction strength
Hip flexion ratio (D/ND)	Ratio	—	Ratio of dominant to non-dominant hip flexion strength
Hamstring ratio (D/ND)	Ratio	—	Ratio of dominant to non-dominant hamstring strength
Hip flexion/hamstring ratio (D leg)	Ratio	—	Ratio of hip flexion to hamstring strength in dominant leg
Hip flexion/hamstring ratio (ND leg)	Ratio	—	Ratio of hip flexion to hamstring strength in non-dominant leg
Hip adduction/abduction ratio (D leg)	Ratio	—	Ratio of hip adduction to hip abduction strength in dominant leg
Hip adduction/abduction ratio (ND leg)	Ratio	—	Ratio of hip adduction to hip abduction strength in non-dominant leg
Combined hip strength (D + ND)	Derived	$Nm·kg^{-1}$ (normalised)	Sum of dominant + non-dominant hip strength for adduction/abduction

Supplementary Material S6. Model Specifications and Hyperparameters

Model	Preprocessing	Feature Selection	Validation Strategy	Hyperparameters (grid/tuned values)	Software
Logistic Regression (elastic-net)	Min–max scaling (nested within training folds)	Symmetrical uncertainty ranking (top 10 predictors retained)	4-fold cross-validation nested within 70:30 train/test split	Penalty: elastic-net, l1_ratio: tuned (0.0–1.0), C (inverse regularisation): default (1.0)	PyCaret (Spyder IDE), Orange v3.4.0, XLSTAT v2014
k-Nearest Neighbours (exploratory)	Min–max scaling (nested)	Same as above	Same as above	k: tuned (3, 5, 7)Distance metric: Euclidean	Same
Support Vector Machine (exploratory)	Min–max scaling (nested)	Same as above	Same as above	Kernel: linear, radialC: tuned (0.1, 1, 10)Gamma: auto	Same

Notes: All preprocessing and tuning were performed strictly within training folds to avoid data leakage. Logistic regression with elastic-net penalty was the primary model; k-NN and SVM were exploratory comparators. Final evaluation was performed on the independent test set (n = 37).

Supplementary Material S7 Confusion Matrices for logistic regression model

Dataset	Actual Injured	Actual Not Injured	Total
Training set (n = 83)			
Predicted Injured	TP = 13	FP = 16	29
Predicted Not Injured	FN = 3	TN = 51	54
Total	16	67	83
Test set (n = 37)			
Predicted Injured	TP = 3	FP = 11	14
Predicted Not Injured	FN = 2	TN = 21	23
Total	5	32	37

Confusion matrices for logistic regression model in the training and independent test sets. TP = true positives; FN = false negatives; TN = true negatives; FP = false positives.

Supplementary Material S8: Final logistic regression model coefficients (player-level outcome)

Predictor	β (SE)	OR	95% CI	p-value
Intercept	0.963 (4.334)	2.62	0.10 – 12.80	0.824
Age (years)	−0.012 (0.050)	0.99	0.90 – 1.09	0.808
BMI (kg/m²)	0.034 (0.136)	1.03	0.79 – 1.35	0.805

Predictor	β (SE)	OR	95% CI	p-value
Previous hamstring injury	−1.283 (0.805)	0.28	0.06 – 1.34	0.111
**Hip abduction (dominant leg)**	−0.200 (0.083)	0.82	0.70 – 0.96	**0.016***
Hip flexion (non-dominant leg)	0.108 (0.067)	1.11	0.98 – 1.27	0.109
Hip adduction ratio (D/ND)	0.939 (1.520)	2.56	0.13 – 50.27	0.536
Hip abduction ratio (D/ND)	−0.414 (1.121)	0.66	0.07 – 5.95	0.712
Hamstring ratio (D/ND)	−0.346 (1.541)	0.71	0.04 – 14.51	0.822
Hip flexion ratio (D/ND)	2.304 (2.251)	10.02	0.12 – 826.22	0.306
Hip flexion/hamstring ratio (dominant leg)	−0.621 (0.947)	0.54	0.08 – 3.44	0.512

Supplementary Material S9. Stability analyses: bootstrap resampling and permutation importance

Analysis type	Settings	Results (test set)
Bootstrap resampling	n = 200 resamples; sampling with replacement from training set; performance evaluated on fixed independent test set	Mean AUC = 0.681; SD = 0.036; 2.5th–97.5th percentiles = 0.600–0.745. Distribution showed modest discrimination, rarely exceeding 0.75.
Permutation importance	n = 50 permutations per predictor; predictors permuted in test set while others held fixed; change in AUC (ΔAUC) recorded	Previous injury: ΔAUC −0.032 ± 0.089 (largest effect). Hip abduction strength (dominant leg): ΔAUC −0.016 ± 0.039 (modest, unstable). Hamstring strength (dominant leg): ΔAUC ≈ 0 (negligible). Age: ΔAUC +0.034 ± 0.041 (noise).
